# Alloys for Die Metal, Etc.

**Published:** 1861-11

**Authors:** B. Wood

**Affiliations:** Indianapolis


DENTAL REGISTER OF THE WEST.

VOL. XV.] NOVEMBER, 1861. [No. 11.

### Original Essays and Communications.

### ALLOYS FOR DIE-METAL, ETC.

BY DR. B. WOOD.

FOR getting up metallic castings, I used, during the first few years of my practice, almost exclusively, an alloy of lead, tin and bismuth, as prepared by my preceptor and brother, Dr. J. S. Wood, to-wit:

*For the die, or cast,*—Tin 8 parts, lead 1 part, bismuth 1 part. This compound is much harder than tin, melts at a lower heat, shrinks little, or practically none, in casting, is tough and strong. It melts at about 330° F. Although generally using a harder and less fusile metal for the first swaging, I find this alloy particularly convenient for taking duplicate dies for finishing. Its tenacity adapts it to cases where teeth are to be represented on the die.

The counter-die, or mould metal, is made by adding to one part of this mixture six parts of lead. The result is harder than lead, and does not yield like it under the blow, presenting a resistance sufficient to drive the plate *up* well against the die. Its shrinkage is but slight. It melts at, say 450° to 460° F. It is designed for use when the dipping process is resorted to. If used at the point of congelation, the plaster cast may be immersed without previous baking, otherwise it should be baked to expel water of crystallization. I frequently place the cast in the metal in a flask for the purpose, then heat the whole again until the cast melts loose, then remove from the fire, pressing it down to its place, and retaining it there by means of a weight on the top, until cool.

I shall now confine myself simply to a description of some of the alloys that I have prepared and found most generally useful, not stopping to speak of those recommended by others—my experiments having had reference to the qualities of hardness, tenacity and fusibility, and the susceptibility of forming perfect castings.

The following formula affords a highly useful alloy, where *toughness*, as well as hardness, is essential;

Tin 16 parts; antimony 1 part; zinc 1 part.

This alloy is much harder than the preceding die-metal, and equals it in tenacity, being suited for any kind of die; it requires a higher temperature for fusion; but it melts sooner than tin, or than the mould-metal above named—from a matrix of which a die may be taken by it with safety. It affords in sand a perfect die, does not shrink, and, whether poured into a sand or metal mould, comes out with a bright, smooth face. It is the best combination of these three metals I was able to produce for the purpose. When, with a view to greater hardness, antimony and zinc were employed in larger proportion; the die cast in sand came out imperfect, presenting fissures, pits or concavities at the point corresponding to the palatine fossa. If tin be used in larger quantity, the alloy is, proportionably softer, and there is corresponding shrinkage. The relation of zinc and antimony in respect to each other may be somewhat varied without sensible modification of the qualities of the compound, but for the best results, the sum of these two metals should hold to the quantity of tin employed the ratio of about one to eight.

This alloy melts readily enough for use with the mould metal as above mentioned. But when dies are first taken with it, a suitable alloy for taking counter-dies from them is obtained by a combination of 5 parts of lead, 2 of bismuth, and 1

of tin ; or, 5 parts lead, 3 or 4 of bismuth, and 1 of tin, will be still more fusible, although harder.

A very hard and most valuable alloy for general use may be had by the use of copper, antimony and tin, to-wit:

Tin 12 parts ; antimony 2 parts ; copper 1 part.

It is not far below zinc in hardness, casts without sensible shrinkage, and makes a perfect and very handsome die, bright and smooth. It is less fusible than the die metal last named, but may be used for taking a die from the first mentioned mould metal, although, melting at nearly the same temperature, this requires care. It will be found of value in connection with lead moulds made by "dipping." It is rather brittle for dies for partial sets representing the teeth, as these are liable to break in removing from the matrix, but it is strong enough for swaging purposes.

A still harder alloy is obtained by using 8 parts of tin, 1 part of copper, and 1 part of antimony. This possesses the general properties of the preceding, but is harder to melt and less *fluid* when melted, and in cooling, crystallization takes place on the surface, roughening the face of the die somewhat to its detriment.

For *fluidity*, an excess of antimony over copper appears to be requisite. For preventing shrinkage, the joint amount of antimony and copper should be to the tin in the ratio of about one to four ;—as for example, 8 parts tin, 1 antimony, 1 copper ; or 10 tin, 1½ antimony, 1 copper ; or 12 tin, 2 antimony, 1 copper.

In combining these metals (which may be done in an ordinary charcoal furnace, as it is by no means requisite to have a temperature capable of melting copper), place the copper in a crucible and bring it to a red heat, then pour in the tin and antimony in a melted state, and cover the whole with charcoal dust to prevent oxidation. The copper will soon liquify, or *dissolve* as it were, and combine perfectly with the other metals without further elevation of temperature. To guard better against volatilization of antimony, which takes place at a high heat, it is well enough to add to the copper at first but half the tin, and when these are combined, add the antimony, and then the remaining tin. This also enables one to conduct the second melting in a larger crucible, or indeed in an iron ladle.

For taking counter-dies, or moulds from dies of the last named alloys, a suitable metal, fusible at about 380° F., is had by a mixture of three parts of lead, one part of bismuth, and one hundreth, or less, part of tin. It is wonderful how small a quantity of tin serves to improve the alloys of lead and bismuth, giving them a white, clear lustre, preventing oxidation, promoting fusibility, in short, producing almost a new metal.

By the use of *cadmium*, we may produce still harder alloys than any of the preceding, possessing in an equal degree every other desirable quality. Thus, 10 parts of tin, 1 part of antimony, 1 part of copper, and 1 part of cadmium produces a compound which has about the hardness of zinc, it casts perfectly, and is nearly all that could be desired, except that, like the last named die metals, it is rather brittle for certain castings, as in the cases before referred to.

Substituted for copper in these combinations, cadmium appears to confer greater hardness and tenacity, (using the latter word in the sense of *toughness*) and up to a certain point, promotes fusibility.

9 parts of tin, 1 part of antimony, and 1 part of cadmium furnish a very hard and tough metal of a compact, homogeneous structure, which casts without shrinkage, forming a perfect die, with a smooth, bright face. It melts at about the melting point of tin.

In the employment of cadmium, care must be taken not to subject it to a heat high enough to volatalize it. To avoid this danger, it is best to unite the other metals first, and then add the cadmium at a heat barely sufficient to melt it. The great objection to the use of this metal is its expensiveness.

A variety of other forms of alloys might be given, some of which, doubtless, would prove useful in special cases, but those given are best adapted to general use, and further than this does not come within the intention of the present writing. Should the results here presented prove of benefit to any in the profession, I shall feel well paid, especially in the assurance that this subject happens to be not one likely to awaken hostility as involving facts in conflict with cherished interests, practices and inclinations ; whereas, when touching upon rival *modes of practice*, a plain record of facts—not swerving from the rigid truth to conciliate interested or prejudiced parties, not taking a middle course between right and wrong, to win the credit of "impartiality"—is too apt to provoke from some source, directly or indirectly, the imputation of "unfairness" —which is rather "poor pay" for one's pains.

Indianapolis, Oct., 1861.

